# AlGaInP LED with low-speed spin-coating silver nanowires as transparent conductive layer

**DOI:** 10.1186/1556-276X-9-670

**Published:** 2014-12-11

**Authors:** Xia Guo, Chun Wei Guo, Cheng Wang, Chong Li, Xiao Ming Sun

**Affiliations:** Photonic Research Lab, Beijing University of Technology, Beijing, 100124 People’s Republic of China; Beijing University of Chemical Technology, Beijing, 100029 People’s Republic of China

**Keywords:** AlGaInP, Light-emitting diodes (LEDs), Low-speed spin-coating, Silver nanowires, Transparent conductive layer

## Abstract

The low-speed spin-coating method was developed to prepare uniform and interconnected silver nanowires (AgNWs) film with the transmittance of 95% and sheet resistance of 20Ω/sq on glass, which was comparable to ITO. The fitting value of *σ*_dc_/*σ*_op_ of 299.3 was attributed to the spin-coating process. Advantages of this solution-processed AgNW film on AlGaInP light-emitting diodes (LEDs) as transparent conductive layer were explored. The optical output power enhanced 100%, and the wavelength redshift decreased from 12 to 3 nm, which indicated the AgNW films prepared by low-speed spin-coating possessed attractive features for large-scale TCL applications in optoelectronic devices.

## Background

Transparent conductive layer (TCL) is crucial for light-emitting diodes (LEDs) which spread the carriers far away from the opaque electrodes to enhance the quantum efficiency and improve the efficiency droop effect [1–3]. Indium-doped tin oxide (ITO) material is widely used in LED field with the sheet resistance *R*_s_ of 10 to 30 Ω/sq and optical transmittance *T* of 90%, which are two important figures of merit (FoM) to facilitate to describe the performance of TCLs [4]. However, due to the scarcity of the element indium in the earth and consequently the soaring prices, recently, nanomaterials, such as carbon nanotubes (CNTs) [5], graphene [6], metal grids [7], and metallic nanowires [8], have attracted great attention as candidates of TCL due to their unique electrical properties, good transparency, and mechanical flexibility. Due to the large inter-junction resistance of CNT film caused by mixture of metallic and semiconducting properties, the sheet resistance of CNT film is 200 to 1,000 Ω/sq [9], which is relatively high compared with that of the ITO film. Graphene has high mobility as well as high transmittance [10, 11]. However, large sheet resistance and obvious degradation of graphene layer under several milliampere current injections restricted its actual application [12].

Random and sparse silver nanowire (AgNW) film [13], which demonstrated superior FoM performances, was regarded as the most promising candidate to replace ITO, due to its low inter-wire junction resistance and low absorption loss [14]. Yi’s group demonstrated solution-processed AgNW films with *T*_550nm_ of 80% and *R*_s_ of 20 Ω/sq [9]. J. N. Coleman’s group sprayed the AgNWs over large areas with *T*_550nm_ of 90% and *R*_s_ of 50 Ω/sq [15]. Such AgNW films as TCLs applied to organic optoelectronic devices were reported. For example, AgNWs film as top electrodes of the organic solar cells or organic LEDs were recently established as a serious alternative to ITO [16–18].

One of the most significant challenges for the AgNW film was to obtain the low sheet resistance and high transmittance at the same time [19]. Besides controlling the diameter and length of the wires, uniform distribution of the wires was another important factor. Conventional drop-coating, spray-coating, and bar-coating processes inevitably caused the solution-based AgNWs self-aggregation after solvent drying process. Poor adhesion to the substrate made the uniform distribution of AgNWs more difficult. Thus, new film coating method as well as substrate material to improve the uniformity of nanowire distribution was required. Filtration coating was developed for preparing AgNW films with *T* of 88% at 550 nm and *R*_s_ of 12 Ω/sq on cellulose nanopaper [20]. Exfoliated clays were utilized for reducing the self-aggregation of nanowires with high solution viscosity on PETs by roll-to-roll coating process with *T* of 97.9% and *R*_s_ of 91.3 Ω/sq [16].

In this paper, a low-speed spin-coating method was developed for uniform AgNW film with the *T* of 94% and *R*_s_ of 20 Ω/sq on glass. The optical output power increased about 100% for AlGaInP LEDs with AgNW film as TCL, and the wavelength redshift decreased from 12 to 3 nm under the current injection of 100 mA due to the uniform carrier injection in the active region.

## Methods

The Ag nanowires were prepared following the reported procedure [21]. In brief, 0.5 g of glucose and 0.1 g of polyvinyl pyrrolidone (PVP) were dissolved in 35 ml of deionized water to form a clear solution. Then, 0.5 ml of freshly prepared 0.1 M aqueous AgNO_3_ solution was added under vigorous stirring. The mixture was transferred into a 40-ml Teflon-sealed autoclave and heated at 140°C for 10 h. After the reaction, the autoclave was allowed to cool in air and the product was purified by 3 to 5 centrifugation/rinsing/redispersion circles. Then, the AgNWs re-dispersed in isopropyl alcohol due to better dispersibility for different concentrations. The AgNW film was fabricated through spin-coating at a speed of 270 rpm, which was much lower than the spin-coating speed of photoresist which was used in the microelectronics field due to the poor adhesion of AgNW solution. The nanowires were stuck on the substrate surface after the solution was spanned out of the substrate. After the spin-coating process, samples with AgNW film was put on the hot plate for 10 min with a temperature of 200°C in order to decrease the nanowire-nanowire contact resistance [22].

## Results and discussion

**Figure 1:**
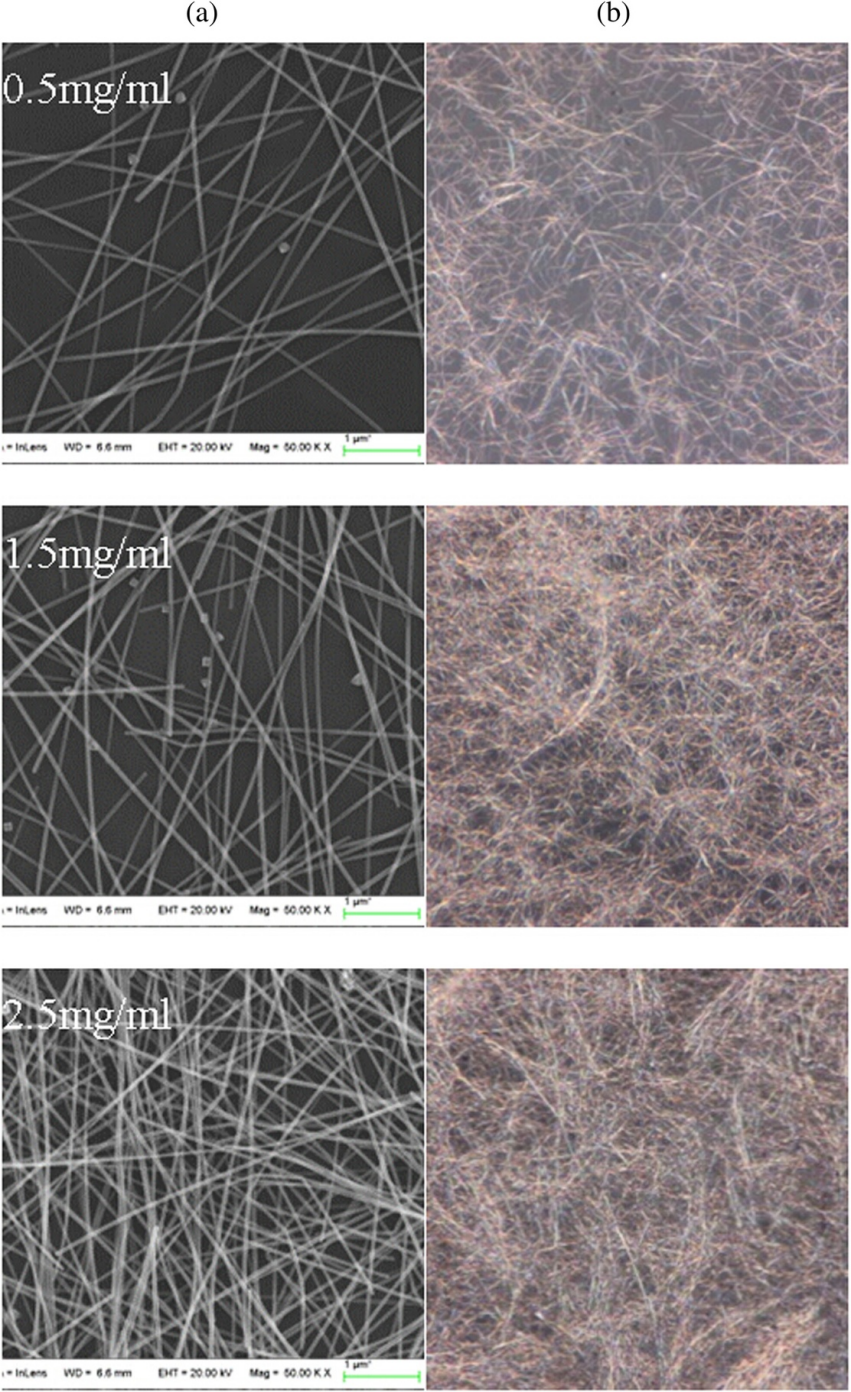
**Scanning electron microscopy (SEM) images (a) and microscopic photographs (b).** Of AgNW films with AgNW concentrations of 0.5, 1.5, and 2.5 mg/ml, respectively. The microscopic photographs, with magnification of × 1,000, displayed the AgNWs were connected with each other over large areas.

**Figure 2:**
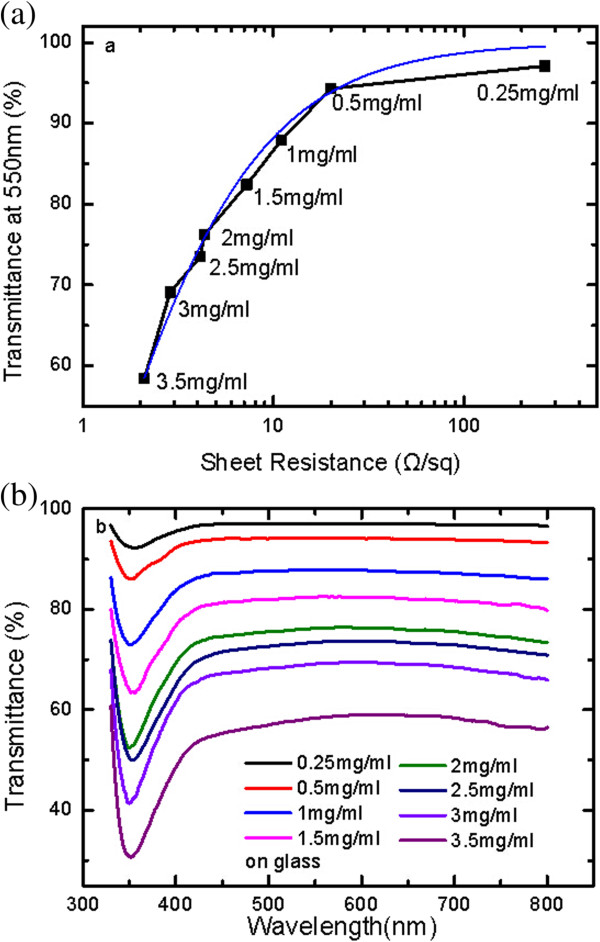
**Optical and electrical performance of AgNW film on glass. (a)** Spectral transmittance at 550 nm as a function of sheet resistance for AgNW films on glass with different concentrations by spin- and drop-coating processes. The fitting curve according to Equation 1 fits the data prepared by spin-coating process well. In each case, the transmittance was evaluated with the bare glass as a reference. **(b)** Spectral transmittance results of AgNW films prepared by spin-coating at 270 rpm with AgNW concentrations from 0.25 to 3.5 mg/ml.

Figure 1a presented the scanning electron microscopy (SEM) images of AgNW films on glass prepared by spin-coating, which was the mostly commonly used in preparing photoresist with large scale in the field of integrated circuits. The concentrations of AgNW solution were 0.5, 1.5, and 2.5 mg/ml, respectively. The rotation speed was closely related with the FoM and was optimized to be 270 rpm, which was far lower than that in the photolithography process due to the poor adhesion of wires to the substrate. From the SEM images, the typical diameter and length of the AgNWs can be measured about 40 nm and 30–50 μm, respectively. But most importantly, the wires connected were with each other and uniformly distributed, which guaranteed low resistance of the AgNW film. Figure 2b showed the corresponding microscopic photographs with magnification of × 1,000, which also displayed the uniform distribution of the AgNW films. By increasing the AgNW concentrations, the AgNW area coverage increased. For the concentration of 2.5 mg/ml, it is hard to find the bare space of substrate under the microscope, which indicated low transmittance.

Figure 2a showed the transparent conductive performances of AgNW film on glass without any pressing treatment after spin-coating. The transmittance was evaluated using a piece of bare glass as reference. The sheet resistances of AgNW film on glass prepared by spin-coating at 270 rpm showed 20Ω/sq with *T*_550nm_ of about 95%. It should be noted that the value (*R*_s_, *T*_550nm_) of (20 Ω/sq, 95%) was comparable with the performance of ITO and was much superior to that of graphene and CNT, which indicated that the AgNW film as transparent conductive layer was capable of applications in the optoelectronic devices, such as LEDs and solar cells, whose performance was sensitive to the power conversion efficiency. Figure 2b showed the spectral transmittance of AgNW film on glass with the AgNW concentrations from 0.25 to 3.5 mg/ml. The transmittance was kept almost flat from about 420 to 800 nm for all the curves, which indicated wide applications in the visible wavelength range. The transmittance decreased with the concentration of AgNW solution due to the nanowire coverage area.

The transmittance and sheet resistance of nanowire film could be expressed as [23]
1

in which *Z*_0_ was the impedance of free space which was equal to 377 Ω. *R*_s_ was the sheet resistance of the nanowire film. *σ*_op_ and *σ*_dc_ were the optical and DC conductivity of the film, respectively. The optical and electrical performance of the film could be evaluated by the ratio of *σ*_dc_/*σ*_op_. High transmittance and low sheet resistance means the large ratio of *σ*_dc_/*σ*_op_. The first criterion for high-performance TCL required the ratio of *σ*_dc_/*σ*_op_ ≥ 35 to achieve the target of T ≥ 90% and *R*_s_ ≤ 100 Ω/sq [14]. In our experiment, the *σ*_dc_/*σ*_op_ of the film prepared by spin-coating was fitted to be 299.3, as shown in Figure 2a, which was close to that of ITO and much larger than that of CNT and graphene [14, 24]. Also, the theoretical prediction fitted all the experimental data very well except the only data with the concentration of 0.25 mg/ml. For comparison, the sheet resistance of AgNW film prepared by conventional drop-coating on glass was 1,000 Ω/sq with *T*_550nm_ of about 95%, and the fitting ratio of *σ*_dc_/*σ*_op_ was only about 31, which indicated that the low-speed spin-coating could improve the FoM of the AgNW film greatly by enhancing the uniformity of nanowire distribution.

To demonstrate its potential of transmittance conductive properties, AlGaInP LEDs with AgNW film as current-spreading layer were fabricated. The reason that demonstration on the AlGaInP LEDs, not GaN-based LEDs, was smaller work function difference of no more than 1.5 eV between the AgNW and GaP, which indicated the feasibility of ohmic contact between metal nanowire and p-doped GaP.

The AlGaInP LEDs were grown on n-GaAs substrate by metal-organic chemical vapor deposition. The details could be found in ref. [25]. In order to study the current-spreading effect of AgNW film, only 500-nm-thick Mg-doped p-GaP window layer with the doping density of 5 × 10^18^ cm^-3^ was grown on top. The 50-, 150-, or 200-nm-thick Au/BeAu/Au with 100 μm diameter was first deposited and then patterned by wet etching as p-type electrode. The AgNW solution with the concentration of 0.5 mg/ml was applied and then stuck on the surface of the LED wafer by Vander Waals force. The chip size was 300 μm × 300 μm in this work.

**Figure 3:**
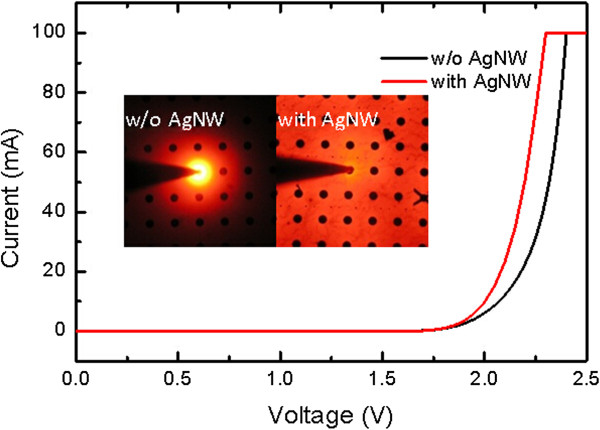
**Current-voltage (** ***I*** **-** ***V*** **) curves of AlGaInP LED with and without AgNWs as current-spreading layer.** The inset showed microscope photographs of LED wafers before dicing under the current injection of 5 mA under the probe station.

Figure 3 showed the current–voltage (*I*-*V*) curves of AlGaInP LED with and without AgNWs as current-spreading layer with the voltage drops of 2.08 and 2.18 V at current injection of 20 mA, which indicated better current spreading. The inset showed the microscope photographs of LED wafers before dicing under the current injection of 5 mA under the probe station. It was obvious that the current-spreading effect was totally different, which echoed the *I*-*V* measurement results. For the devices without AgNWs, the emission was localized around the electrode, which indicated the carriers transport laterally with limited distance. While for the devices with AgNW film, the whole wafer was lighting up, which demonstrated the excellent capability of lateral carrier transport of AgNW film.

**Figure 4:**
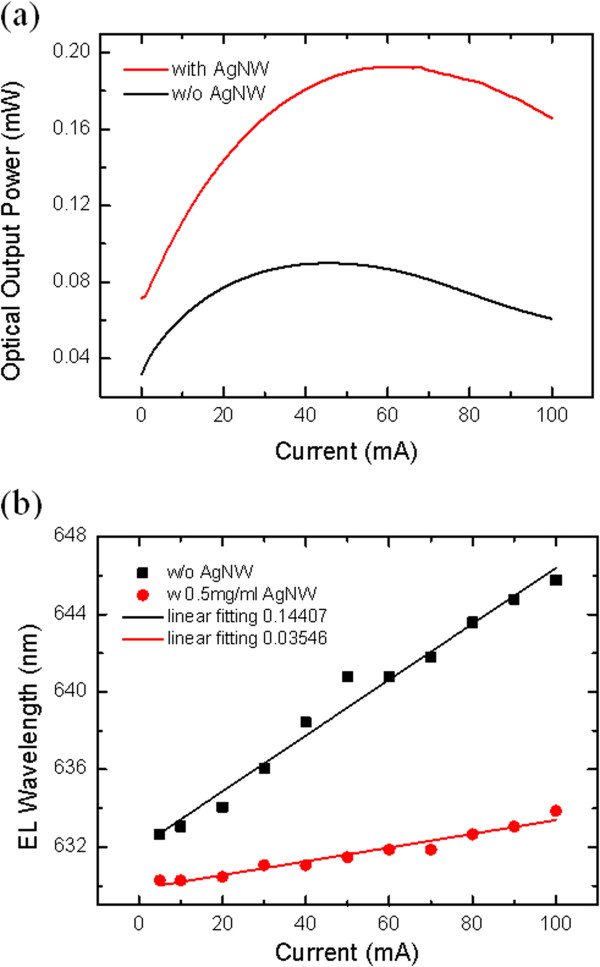
**Dependence of optical output power and peak wavelength. (a)** The dependence of optical output power on the current injection of LED chips with and without AgNW film as current-spreading layer. **(b)** The dependence of peak wavelength of AlGaInP LEDs with and without AgNW film on the current injection.

Figure 4a demonstrated the optical output power on the current injection of LED with and without AgNW film as TCL. The optical output power and the linearity of LED with AgNW film were much better than that of without AgNW film. At 20 mA, the optical output power of LED with AgNW film was two times of that of without AgNW film. As we known, the optical output power improved only 30% if ITO as TCL on LEDs [26, 27]. Nano or microstructures, such as photonic crystal and surface roughness, could only improve the optical output power about 10% to 30% [28]. The current value corresponding to the maximum optical output power was 60 and 40 mA, respectively, with and without AgNW film, which indicated the better thermal performance. The peak wavelength was 630 and 635 nm, respectively, according to the electroluminescence spectra of LEDs with and without AgNW film at 20 mA. The wavelength redshift was another important criterion to characterize the current-spreading effect, and AlGaInP material was very sensitive to the temperature. Figure 4b demonstrated the wavelength redshift measurement results, in which the dots were the measurement data and the line was the linear fitting of the data. The wavelength redshift was 3 and 12 nm for LEDs with and without AgNW film, respectively, which verified the optical output power results.

The obvious improvement of LED’s optical output power and thermal performance, we believe, not only due to the high FoM of AgNW film but also due to the current injection in different ways. The network of nanowires on the LED formed an equipotential connection after biasing. All the nanowires uniformly distributed on the surface of the LED injected the carriers at the same time with lowered current density, just like water from a shower head. Compared with the current injection from the ohmic contact electrode which usually located at the center of the device, the current density distribution in the quantum wells from the nanowire film will be more uniform, which decreased the current crowding and heat generation.

## Conclusions

In summary, low-speed spin-coating method was demonstrated to prepare uniform and interconnected AgNW film with the transmittance of 95% and sheet resistance of 20 Ω/sq on glass, which was comparable to ITO. The fitting value of *σ*_dc_/*σ*_op_ of around 300 was attributed to the spin-coating process. Advantages of this solution-processed AgNW film on AlGaInP LEDs as TCL were explored. The optical output power enhanced 100% and the wavelength redshift decreased four times, which indicated the AgNW films prepared by low-speed spin-coating-possessed attractive features for wide TCL applications in optoelectronic devices.
